# Patients with Multiple Functional Gastrointestinal Disorders (FGIDs) Show Increased Illness Severity: A Cross-Sectional Study in a Tertiary Care FGID Specialty Clinic

**DOI:** 10.1155/2020/9086340

**Published:** 2020-01-28

**Authors:** Sabrina Berens, Felicitas Engel, Annika Gauss, Jonas Tesarz, Wolfgang Herzog, Beate Niesler, Esther Stroe-Kunold, Rainer Schaefert

**Affiliations:** ^1^Department of General Internal Medicine and Psychosomatics, University of Heidelberg, Im Neuenheimer Feld 410, D-69120 Heidelberg, Germany; ^2^Department of Gastroenterology, Infectious Diseases and Intoxications, University of Heidelberg, Im Neuenheimer Feld 410, D-69120 Heidelberg, Germany; ^3^Institute of Human Genetics, Department of Human Molecular Genetics, University of Heidelberg, Im Neuenheimer Feld 366, D-69120 Heidelberg, Germany; ^4^Department of Psychosomatic Medicine, Division of Internal Medicine, University Hospital Basel, Hebelstrasse 2, CH-4031 Basel, Switzerland; ^5^Faculty of Medicine, University of Basel, Klingelbergstrasse 61, CH-4056 Basel, Switzerland

## Abstract

**Objectives:**

Overlaps between different functional gastrointestinal disorders (FGIDs) are common. However, little is known about the impact of this overlap on patients' health status. This study is aimed at analyzing the differences between patients with multiple as compared to one single FGID.

**Methods:**

A retrospective, cross-sectional study was conducted with patients presenting to a tertiary care FGID specialty clinic between 06/2012 and 01/2015 (*n* = 294). They were characterized primarily according to their GI symptom severity (IBS-SSS) and secondarily to their physical as well as psychosocial symptom burden, quality of life, health care utilization, and work-related impairment. Differences between patients with >1 vs. 1 FGID were analyzed.

**Results:**

Of the 294 patients, 92.2% fulfilled the Rome III criteria for any FGID, and 48.0% had >1 FGIDs. FGID patients had a median age of 38 [23.0] years; 72.0% were female. Median GI symptom severity (IBS-SSS) scores were 339 [126] and 232 [163] in patients with >1 and 1 FGID, respectively (*p* < .001). Furthermore, patients with >1 FGIDs had higher general somatic symptom severity, higher illness anxiety, lower quality of life, and more work-related impairment. Almost no differences were found regarding their somatic as well as mental comorbidities.

**Conclusions:**

Multiple FGIDs are associated with an increased risk for complicated courses of illness as reflected in higher GI and somatic symptom severity, as well as stronger psychosocial and diet- and work-related impairment. Stepped and interdisciplinary models of care including psychosocial expertise and dietary advice are needed, especially for patients with multiple FGIDs.

## 1. Introduction

Functional gastrointestinal disorders (FGIDs) manifest as characteristic combinations of troublesome symptoms arising from the gastrointestinal (GI) tract [1]. They are usually classified according to the Rome criteria, which are based on patients' self-reported symptoms [2]. FGIDs are highly prevalent; the most frequent FGIDs are irritable bowel syndrome (IBS) and functional dyspepsia (FD), affecting 8.1% (IBS) [3] and 11.5-14.7% (FD) [4] of the general population, respectively.

There is considerable overlap between the different FGIDs [5, 6]. Previous studies reported the following correlates of multiple FGIDs: higher GI symptom severity [7], somatization [8], higher depression and anxiety rates [9, 10], lower quality of life [11], and increased physician consultations [12]. Therefore, patients with multiple FGIDs are supposed to be more severe cases. The clinical severity of FGIDs is estimated to be mild in 40%, moderate in 35%, severe/complicated in 20%, and very severe in 5% of affected patients [13, 14]. As illness severity increases, psychosocial variables gain more relevance [14]. More severe courses are associated with multiple and persistent somatic symptoms, numerous psychosocial stressors, high emotional distress, disproportionate illness anxiety, high functional impairment, frustrating physician-patient relationships, and dysfunctional health care utilization [13]. High levels of psychological as well as intestinal and extraintestinal somatic comorbidities further contribute to the burden of FGIDs [15], which often considerably impair patients' quality of life (QoL) [16] and lead to high health care costs [17].

Patients with a mild course usually can sufficiently be managed by primary care physicians (PCPs) and/or gastroenterologists. In patients with a more severe/complicated course, primary care or standard gastroenterologic treatment often does not provide adequate symptom relief [18, 19], and the patients frequently are dissatisfied with standard medical care [11]. For this latter group of patients, a severity-stepped, interdisciplinary, and biopsychosocial approach including psychosomatic support is recommended. To meet these needs, an interdisciplinary FGID specialty clinic was developed and implemented at the Department of General Internal Medicine and Psychosomatics of Heidelberg University Hospital [20].

This study is aimed at evaluating differences between patients seen in this specialty clinic with >1 FGID vs. 1 FGID according primarily to their GI symptom severity and secondarily to further physical and psychosocial symptom burden, quality of life, health care utilization, and work-related impairment. This should help to identify more severe courses of illness and support the development of appropriate stepped and shared care models.

## 2. Methods

### 2.1. Study Design

We conducted a retrospective, observational study in a tertiary care setting including all 294 patients who visited our outpatient FGID clinic at the Department of General Internal Medicine and Psychosomatics at Heidelberg University Hospital between 06/2012 and 01/2015. Patient management and data collection took place under conditions of routine care. According to the national regulations, need for informed consent was deemed unnecessary. A quasiexperimental study was conducted to compare patients with >1 vs. 1 FGID according primarily to their GI symptom severity and secondarily to further physical and psychosocial symptom burden, quality of life, health care utilization, and work-related impairment. The study was approved by the ethics committee of Heidelberg University (S-641/2015).

### 2.2. Measurements

Based on current guidelines and evidence [19, 21, 22], the model of care for our FGID clinic employs a simultaneous approach by considering somatic as well as psychosocial factors of illness in assessment and therapy [20, 23]. Additionally, recommendations of phenotyping IBS patients for large-scale studies were picked up [24]. The following physical and psychosocial features were assessed by a set of general and FGID-specific questionnaires that every patient completed before they were seen by a physician. Additional information referring to somatic comorbidities was supplemented by the physician letter.

### 2.3. FGID Diagnoses

Patients were diagnosed with a selection of FGIDs according to Rome III criteria: IBS (including IBS subtypes diarrhea/constipation/mixed/unspecified), FD, functional bloating, functional constipation, and functional diarrhea [2]. For analyses, they were classified into two groups (>1 FGID vs. 1 FGID), whereby not every combination of FGID diagnoses was possible: according to the Rome III diagnostic criteria, functional bloating, functional diarrhea, and functional constipation were excluded, if IBS was the main diagnosis. Furthermore, functional bloating was excluded if another FGID had been diagnosed [2].

### 2.4. Sociodemographic Characteristics

Our psychosomatic basis documentation questionnaire (Psy-BaDo) was used to collect information about age, gender (female/male), nationality (German/other), marital status (living with a partner; yes/no), educational level (ISCED ≤2/>2), professional life (paid employment/disability pension/old-age pension), duration of GI symptoms, medical appointments during the last 4 weeks (0, 1, 2, ≥3 appointments), psychotherapeutic treatment (ever/in the past/currently), and use of medication for anxiety, depression, or stress (yes/no) [25].

### 2.5. GI Symptom Severity and Further Physical Features

- GI symptom severity was categorized using the irritable bowel severity scoring system (IBS-SSS) [26]. The following are the part one of the IBS-SSS scores (1) severity, (2) frequency of abdominal pain, (3) severity of abdominal distension, (4) dissatisfaction of bowel movements, and (5) the interference with life, with a 100-point scale (0, none, and 100, worst) for each of the five questions (total range 0-500). The severity score is graded as low (<75), mild (75-174), moderate (175-300), and severe (>300). As shown by Kanazawa et al. [27], the symptom questions of the IBS-SSS are appropriate for characterizing the severity of symptoms in IBS as well as the severity of FD with the possible exception of the bowel dissatisfaction item
- Stool frequency (minimum/maximum) was assessed using the respective items of part two of the IBS-SSS [26]
- Stool consistency was assessed using the Bristol Stool Form Scale (BSFS) and classified into hard (1-2), normal (3-5), loose (6-7), and changing (8) (multiple answers possible) [28]
- Somatic symptom severity (SSS)/somatization was measured using the somatic symptom scale-8 (SSS-8, range 0-32) [29] and the SSS scale of the general patient health questionnaire (PHQ). The latter was used as PHQ-15 (range 0-30) and, excluding the three GI items, as PHQ-12 (range 0-24) [30, 31]. Additionally, the categorical diagnosis of somatoform syndrome was made [32]
- Somatic comorbidities: comorbid somatic diagnoses that could produce GI complaints were captured within the physician letters. For this study, the following reliable diagnostic information was assessed: food intolerances (lactose intolerance, fructose malabsorption, histamine intolerance, sorbitol intolerance, and celiac disease), food allergies, gastritis, gastroesophageal reflux, Helicobacter pylori (current and status post), problems of the biliary system (gallstones, cholecystitis, postcholecystectomy syndrome, sphincter of Oddi dyskinesia), thyroid diseases (hyperthyroidism and hypothyroidism), diverticulosis, bile acid malabsorption, intestinal dysbiosis, gastrointestinal motility disturbances (e.g., slow transit constipation), pancreatic diseases, gastrointestinal cancer, inflammatory bowel diseases, neurological diseases, and previous operations

### 2.6. Psychosocial Features

- Rome III, Psychosocial Alarm Questionnaire: items for abuse (lifetime history of emotional/physical/sexual abuse; yes/no) and suicidal tendency (dichotomized; never/at least occasional) were used [1]
- FGID-specific QoL was assessed using the Functional Digestive Disorders Quality of Life Questionnaire (FDD-QoL, range 0-100) with 8 subscales (activities/anxiety/diet/sleep/discomfort/health perception/disease coping/stress) [33]
- Depression was measured using the PHQ-9 depressive symptom severity scale (range 0-27) [34]; additionally, the categorical diagnoses of major depressive syndrome and other depressive syndromes were used [32]
- Generalized anxiety and other anxiety syndromes were assessed using the GAD-7 (range 0-21) [35]; additionally to the dimensional measure, the categorical diagnoses of general anxiety syndrome and other anxiety syndromes were used [32]
- Panic was measured using the 5-item PHQ panic module and evaluated with the categorical algorithm validated and recommended by Löwe and colleagues [32]
- Eating disorders were assessed categorically based on clinical diagnostics and included anorexia nervosa, bulimia nervosa, binge eating, and other eating disorders. Information was supplemented out of the physician letter
- Illness anxiety was measured with the brief Whitley Index-7 (WI-7, range 0-28) [36]. The score was dichotomized and a total score ≥ 4 served as the cut-off indicating relevant illness anxiety [37, 38]
- General QoL was measured with the 36-item Short Form 36 Health Survey (SF-36), generating composite scores (range 0-100) for physical (PCS) and mental health (MCS) [39]
- Work-related impairment: the information, how many weeks the patients were absent from work (absenteeism) or at work suffering due to their GI symptoms (presenteeism) during the last year, was taken from part two of the IBS-SSS [26]

### 2.7. Statistical Analyses

The patient population was characterized using descriptive analyses. Metric variables were reported as total values or means with standard deviation (SD) if they were normally distributed; otherwise, they were reported as median with interquartile range [IQR]. Patients were classified into the two groups (>1 FGID vs. 1 FGID) according to the Rome III criteria. Comparisons between patients with >1 vs. 1 FGID were calculated using the chi-square tests for frequencies and *t*-tests or Mann–Whitney *U* tests for metric variables. Missing values were replaced using mean value imputation, if their frequency was below 20% [40]. The power of the sample (2 groups 140/131) enables to find significant results to effects with *d* > 0.3 (G∗Power calculation with alpha = 0.05 and power = 0.8). All statistical analyses were conducted using SPSS 22/23.

## 3. Results

### 3.1. Referral

Of the 294 patients seen in our tertiary care FGID clinic between 06/2012 and 01/2015, 66.3% were referred by PCPs, 8.8% internally, 5.8% by resident gastroenterologists, and 2.7% by others (e.g., internists, psychiatrists); additionally, 16.3% presented on their own initiative.

### 3.2. FGIDs, Subtypes, and Overlap

Among the 294 analyzed patients (71.8% female, median age 38 years [23]), 92.2% (271) fulfilled the Rome III criteria for any FGID [20]. For the identified FGIDs and the diagnostic overlap, see Table 1. IBS subtypes (*n* = 220) were distributed as follows: 44.1% diarrhea (IBS-D), 40.5% mixed (IBS-M), 11.8% constipation (IBS-C), and 2.3% unspecified (IBS-U). Overall, 52.0% (141/271) of all FGID patients had 1 FGID, 48.0% (130/271) had 2 FGID diagnoses, and no patients had >2 FGID diagnoses. Of all IBS patients, 57.3% also met the FD criteria; of all FD patients, 82.4% also fulfilled the IBS criteria. The diagnoses were distributed as shown in Table 1.

All overlapping diagnoses are shown. According to Rome III diagnostic criteria functional bloating, functional diarrhea and functional constipation were excluded, if IBS was the main diagnosis. Furthermore, functional bloating was excluded, if any other FGID had been diagnosed [2] (total *n* = 271).

### 3.3. Sociodemographic Characteristics

Among the 271 FGID patients, the median age was 38 years [IQR: 23]. The sample was 72.0% female and 94.0% German. Overall, 56.1% of the patients reported living with a partner; 46.5% had a low educational level (ISCED < 2) [41]; and 53.1%, 11.8%, and 3.0% reported paid employment, an old-age pension, and a disability pension, respectively. Regarding sociodemographic characteristics, no significant differences were found between patients with >1 vs. 1 FGID; see Table 2.

### 3.4. Gastrointestinal Symptom Severity and Further Physical Symptom Burden

**Figure 1:**
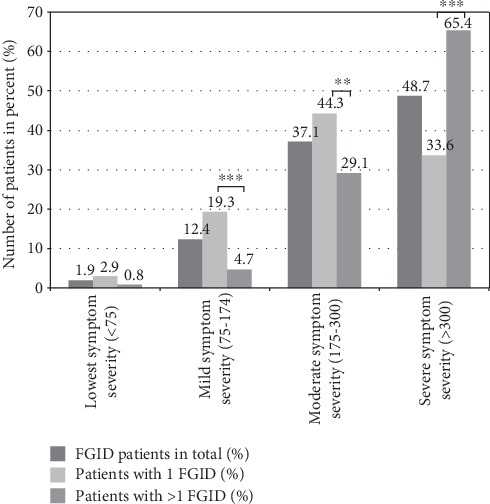
Differences in GI symptom severity (IBS-SSS, range 0-500) in FGID patients (total FGID patients (effective *n* = 267) vs. patients with 1 FGID (effective *n* = 140) vs. patients with >1 FGID (effective *n* = 127)); ^∗^*p* ≤ 0.05; ^∗∗^*p* ≤ 0.01; ^∗∗∗^*p* ≤ 0.001.

GI symptom severity was significantly (*p* < 0.001) higher in patients with >1 FGID (IBS-SSS 339 vs. 232).  Figure 1 shows the differences broken down into the severity categories of the IBS-SSS. General somatic symptom severity was significantly (*p* = 0.008) higher in patients with >1 FGID (PHQ-15 14 vs. 12). Stool consistency (BSFS) was distributed in FGID patients as follows: 70.1% loose (6-7), 47.4% normal (3-5), 33.8% hard (1-2), and 64.1% changing (8); no differences were found between patients with >1 vs. 1 FGID. FGID patients suffered from GI symptoms for a median duration of 4 years (Table 2).

### 3.5. Somatic Comorbidities

**Figure 2:**
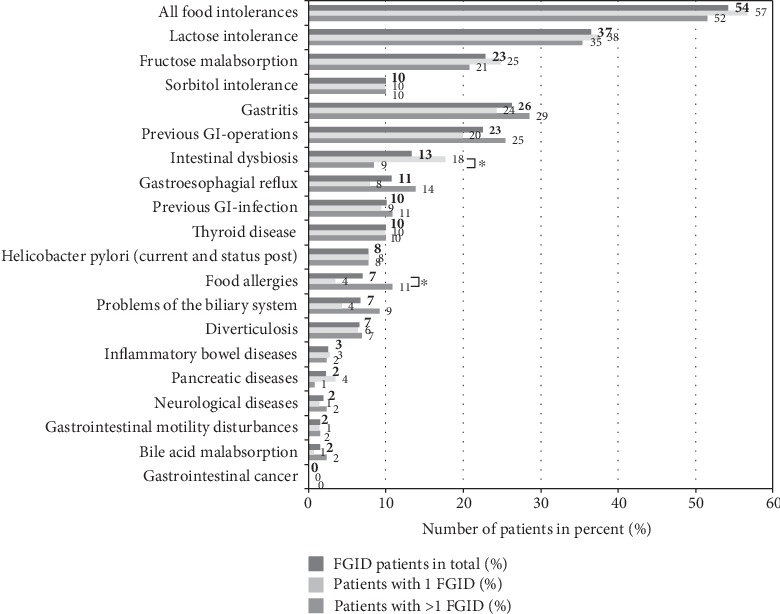
Somatic comorbidities in FGID patients (total FGID patients (effective *n* = 271) vs. patients with 1 FGID (effective *n* = 141) vs. patients with >1 FGID (effective *n* = 130)); ^∗^*p* ≤ 0.05.

Overall, 87.3% of the FGID patients had any somatic comorbidity.  Figure 2 shows the somatic comorbidities in descending order of frequency. The most frequent somatic comorbidities were food intolerances (54.2%), gastritis (26.3%), and previous GI operation (22.5%).

### 3.6. Psychosocial Features and QoL

Psychosocial burden is shown in Table 2. Among all FGID patients seen in our tertiary care specialty clinic, a total of 75.2% had any mental comorbidity; 65.1% had a somatoform syndrome, 41.2% had any depressive syndrome, 18.6% had any anxiety syndrome, and 10.0% had an eating disorder; 17.7% had a lifetime history of abuse, and 20.1% had recent suicidal ideas—all with no significant differences between patients with >1 vs. 1 FGID. While no differences were found according to depression or anxiety, illness anxiety was significantly (*p* = 0.022) higher in patients with >1 FGID (WI-7 13 vs. 10). The following dimensions of GI-specific and general health-related QoL were significantly poorer in patients with >1 FGID: total GI-specific QoL, daily activities, diet, sleep, discomfort, health perception, and total physical QoL.

### 3.7. Health Care Utilization and Illness Behavior

Within one year, FGID patients had a median of 2.0 [IQR: 2.0] appointments in the FGID clinic (Table 3). The use of mental health care was high with no substantial difference between patients with >1 vs. 1 FGID: among all FGID patients, 30.3% visited our psychosomatic outpatient clinic. More than half of the patients reported current or previous psychotherapeutic treatment, and 30.1% currently underwent psychopharmacotherapy.

All FGID patients reported less than 10% absenteeism from work due to GI symptoms, but greater presenteeism (median of 25 weeks). Patients with >1 FGIDs showed significantly greater work-related impairment, resulting in three times more absenteeism and presenteeism.

## 4. Discussion

We analyzed the differences between patients with multiple as compared to one single FGID presenting to our interdisciplinary tertiary care FGID specialty clinic. The patient population exhibited a substantial physical as well as psychosocial burden, especially in patients with >1 FGIDs. As compared to patients with a single FGID, patients with multiple FGIDs reported significantly higher GI symptom severity, higher somatic symptom severity, illness anxiety, lower GI-specific and physical QoL, and greater work-related impairment.

Overall, 92.2% of the patients seen in our tertiary care FGID clinic were diagnosed with at least one FGID and 48.0% with more than one. A less specialized tertiary care gastroenterology clinic in Ireland with a special interest in FGIDs reported only 41.2% of patients had any FGID diagnosis. [42] The number of overlapping FGIDs was similar to other tertiary care studies, which found overlapping FGIDs in 49.6 to 56.4% [5, 43, 44].

Beyond the bare numbers of FGIDs, our patients showed a very high symptom severity: the highest IBS-SSS severity degree > 300 was reported by 33.6% of patients with 1 FGID vs. 65.4% of patients with >1 FGIDs (*p* < .001); altogether, 48.7% of our FGID patients reported the highest IBS-SSS severity level. These percentages in our FGID specialty clinic are higher than the rate reported by Drossman et al. [14]: in Drossman et al. [14], a total of 1,966 IBS patients meeting Rome III criteria (83% female, mean age 49 years, 60% married/cohabiting, 91% White, 78% United States/Canada, mean of 6.6 years of illness) 25% reported the highest IBS-SSS severity degree > 300 [14]. Consistent with our results, a higher GI symptom burden has been found to be associated with more physical complaints, severe emotional symptoms, and a high degree of work impairment [13, 14]. Comparable to our sample with 72% females, women have been shown to be more often suffering from complicated courses of illness [14]. Overall, the high symptom burden shows the high specialization of our FGID clinic with patients already cascaded through the health care system. Despite the high specialization of our center, 16.0% of the patients came on their own initiative. This reflects the high demand and present lack of specific care offers for FGID patients.

Besides the GI symptom severity, also the general somatic symptom severity was significantly increased in multiple FGIDs—even without GI items (PHQ-12). This could indicate a general trend of increased somatization in these patients. Somatization seems to be a risk factor for multiple FGIDs: a current population-based mailing study with a total of 3,548 people found the higher the somatization score, the more likely the overlap of FGID complexes suggesting a dose-response like effect [8].

Otherwise, a higher somatic symptom score could be due to more somatic comorbidities in patients with >1 FGID. A study from a FGID tertiary care referral clinic in Ireland with a total of 1,090 patients reported patients with overlapping FGID syndromes had significantly more non-GI comorbid conditions than patients with a single FGID (*p* < 0.03) [42]. Moreover, 80% of our FGID patients indicated somatic comorbidities, which may have contributed to abdominal discomfort (see Figure 2). According to the German S3 guidelines on IBS, these comorbidities should only exclude FGIDs if they fully explain the GI symptoms [22].

Among our FGID patients, the most frequent somatic comorbidities were food intolerances (54.2%), gastritis (26.3%), and previous GI operation (22.5%). Food intolerances generally affect 20-25% of the general population, but 50-70% of IBS patients [13]. The most frequent food intolerances in IBS patients are fructose malabsorption and lactose intolerance [45] and if we follow the results of Goebel-Stengel [45], our findings still underestimate their frequency. Furthermore, patients with >1 FGID showed more self-reported food allergies and poorer symptom-related QoL with regard to the diet subscore. This is consistent with previous results, showing that patients reported more food items to provoke GI symptoms with increasing IBS symptom severity [46]. These results indicate the importance of including dietary advices within multimodal treatment [20]. Besides of food intolerances, 26% of FGID patients showed comorbid gastritis in our study. Gastritis is one of the most common GI-specific comorbidities in patients with FGID [42, 47]; e.g., Whitehead et al. found 31% comorbid gastritis in IBS, what is comparable to our results [47]. In our study, 22% of FGID patients had previous GI operations. It is known that IBS patients undergo disproportionately high rates of abdominal surgery [48], mainly appendectomy, hysterectomy, and cholecystectomy, and also laparotomy [49]. Within the treatment of FGID patients, it is an important aim to protect them from unnecessary and harmful surgery that often leads to iatrogenic chronification.

The psychosocial burden of our sample was considerable. A recent review reported the following general mental comorbidity rates in FGIDs: [50] 30% depression, 30-50% anxiety disorders, and 15-38% suicidal ideation. Compared to a tertiary care center in New Delhi, India [51], we found similar total mental comorbidity, but more somatization and less depression and anxiety. Also other studies found significantly higher levels of depression and anxiety in patients with >1 FGID [10]. Maybe due to the generally mild depressive and anxiety symptoms in our sample, we could not support this finding, except for illness anxiety. The mild anxiety and depressive symptoms may be due to the more psychosomatically minded doctors referring to our clinic, potentially contributing to a relatively intensive psychosocial pretreatment: 53.1% of our patients reported current or previous psychotherapy and 30.1% had current psychopharmacotherapy, compared to 7.6% of patients with psychopharmacotherapy in the Indian study [51]. However, the psychosocial pretreatment did not seem to adequately decrease illness anxiety, GI, and somatic symptom severity. This may be due to insufficiently treated aspects of physical complaints, too little FGID-specific psychosocial treatments, or the somatic symptoms being especially tenacious. Also with regard to the poorer QoL of patients with >1 FGID, treatments seem to require better coping strategies in dealing with physical symptoms and illness anxiety.

Regarding work-related impairment, presenteeism may have critical economic impact. Our FGID patients reported a median of 25 weeks of suffering from GI symptoms at work, with only two weeks absenteeism. In the pertinent literature, estimates of time lost through presenteeism vary widely, with patients reporting between 2% and 32% of their working week lost due to IBS, depending on symptom severity [52].

Overall, most of the FGID patients in our study had somatic comorbidities and many of them had a high psychosocial burden. A greater somatic symptom severity and an increased number of intervening psychosocial and other comorbidities influence clinical presentation of FGID patients and require adapted treatments [53]. This underlines on the one hand the importance of simultaneous diagnostic assessment of physical as well as psychosocial issues. On the other hand, it calls for multimodal, interdisciplinary approaches and specific psychotherapeutic strategies for FGID patients with more severe courses [20].

The strength of our study is that it presents a naturalistic, routine-based, and broad picture of the patient population of a tertiary care FGID clinic. Additionally, this study picked up relevant recommendations of phenotyping IBS patients for large-scale studies by assessing multiple FGIDs, GI symptom patterns and severity, diet-specific aspects, and psychological comorbidity [24]. Our study has limitations. It is not representative for FGID patients in general, as referred patients have cascaded through care before arriving in our specialized center. The data have been captured using a cross-sectional design; this limits the observation to one time point during their disease, just at the time point when a referral was initiated, which may possibly lead to overestimation of the quantity and quality of symptoms. The study was retrospective and observational; consequently, causal inferences cannot be drawn. Due to the retrospective nature of our study, there may also have been a loss of data; however, the effective *n* were high and missing values small for all items except for work-related impairment; so the latter needs to be considered with caution. Due to routine care requirements, only a shortened Rome III questionnaire could be used, which did not include all FGIDs; therefore, the number of overlapping FGIDs may be underestimated. As many features of FGID patients were explored, significant results must be considered with caution; because of the exploratory nature of this study, we made no adjustment to the significance level to account for multiple testing.

## 5. Conclusions

Compared to patients with 1 FGID, patients with >1 FGIDs—in this sample mostly IBS plus FD—experienced higher GI as well as general somatic symptom severity, higher illness anxiety, lower quality of life, and more work-related impairment. Our results suggest that multiple FGIDs reflect a more complicated course of illness. Therefore, clinicians should notice the number of FGIDs as a considerable marker of illness severity. As patients with multiple FGIDs show a high somatic as well as psychosocial illness burden, a biopsychosocial model of care is needed that offers multimodal treatment adapted to individual patient needs. In future research, common and differential mechanisms between multiple FGIDs should be addressed.

## Figures and Tables

**Table 1:** Distribution of diagnostic overlap between the different FGIDs.

Diagnoses	IBS	FD	Functional bloating	Functional constipation	Functional diarrhea
Overall, *n* (%)	220 (81.2)	153 (56.5)	17 (6.3)	7 (2.6)	4 (1.5)
Single, *n* (%)	94 (42.7)	23 (15.0)	17 (100.0)	4 (57.1)	3 (75.0)
Comorbid FD, *n* (%)	126 (57.3)	—	—	3 (42.9)	1 (25.0)
Comorbid IBS, *n* (%)	—	126 (82.4)	—	—	—

**Table 2:** Sociodemographic, physical, and psychosocial features of FGID patients.

	Effective *n*	FGID patients in total (*n* = 271)^∗^ [20]	Patients with 1 FGID diagnosis (*n* = 141)	Patients with >1 FGID diagnoses (*n* = 130)^∗∗^	*p* value
Sociodemographic characteristics					
Age—years^2^	271	38.00 [23.00]	39.00 [24.00]	35.50 [24.00]	0.548^c^
Gender—female, *n* (%)	271	195 (72.00)	96 (68.09)	99 (76.15)	0.140^b^
Nationality—German, *n* (%)	265	249 (94.00)	130 (95.59)	119 (92.25)	0.254^b^
Marital status—with a partner, *n* (%)	271	152 (56.10)	81 (57.45)	71 (54.62)	0.639^b^
Educational level—ISCED ≤2, *n* (%)	271	126 (46.50)	65 (46.10)	61 (46.92)	0.892^b^
Paid employment, *n* (%)	271	144 (53.10)	77 (54.61)	67 (51.54)	0.613^b^
Old-age pension, *n* (%)	271	32 (11.81)	15 (10.64)	17 (13.08)	0.534^b^
Disability pension, *n* (%)	271	8 (3.00)	3 (2.13)	5 (3.85)	0.486^b^
Gastrointestinal symptoms					
Symptom duration—months^2^	255	48.00 [101.00]	48.00 [76.00]	48.00 [101.00]	0.548^c^
Symptom severity (IBS-SSS)^2^	267	290.00 [169.00]	232.00 [162.50]	339.00 [126.00]	**<0.001** ^c^
Stool consistency					
Loose (BSFS 1, 2), *n* (%)	266	190 (71.43)	100 (72.46)	90 (70.31)	0.698
Normal (BSFS 3, 4), *n* (%)	266	126 (47.37)	64 (46.38)	62 (48.44)	0.737
Hard (BSFS 5, 6, 7), *n* (%)	266	86 (32.33)	45 (32.61)	41 (32.03)	0.920
Any mental disorder, *n* (%)	250	188 (75.20)	92 (71.88)	96 (78.69)	0.212^b^
Somatization (PHQ-15)					
Any somatoform syndrome, *n* (%)	255	166 (65.10)	79 (60.77)	87 (69.60)	0.139^b^
PHQ-15 (range 0-30)^2^	250	13.00 [7.00]	12.00 [6.41]	14.00 [5.98]	**0.008** ^c^
PHQ-12 (range 0-24)^2^	250	8.00 [5.71]	7.00 [5.00]	8.00 [5.00]	**0.028** ^c^
SSS-8 (range 0-32)^2^	261	13.00 [9.07]	11.43 [9.00]	14.00 [10.00]	**0.001** ^c^
Depression (PHQ-9)					
Any depressive syndrome, *n* (%)	262	108 (41.22)	56 (41.48)	52 (40.94)	0.930^b^
Major depressive syndrome, *n* (%)	263	66 (25.10)	36 (26.67)	30 (23.43)	0.546^b^
Other depressive syndrome, *n* (%)	263	42 (15.97)	20 (14.81)	22 (17.19)	0.600^b^
PHQ-9 (range 0-27)^2^	269	9.00 [7.37]	8.00 [8.00]	9.00 [7.50]	0.189^c^
Anxiety (GAD-7; PHQ panic module)					
Any anxiety syndrome, *n* (%)	258	48 (18.60)	24 (18.18)	24 (19.05)	0.858^b^
Other anxiety syndrome, *n* (%)	259	30 (11.58)	15 (11.36)	15 (11.81)	0.910^b^
Panic syndrome, *n* (%)	263	27 (10.27)	15 (11.11)	12 (9.38)	0.643^b^
GAD-7 (range 0-21)^2^	267	7.00 [8.00]	7.00 [8.50]	7.00 [8.00]	0.725^c^
Other psychosocial features					
Illness anxiety (WI-7; range 0-28)^2^	264	11.00 [10.00]	10.00 [10.00]	13.00 [10.25]	**0.022** ^c^
Any eating disorder, *n* (%)	261	27 (10.00)	16 (11.35)	11 (8.46)	0.428^b^
Abuse (Rome III), *n* (%)	253	48 (18.97)	19 (14.50)	29 (23.77)	0.060^b^
Suicidality (Rome III), *n* (%)	259	52 (20.07)	25 (18.80)	27 (21.43)	0.597^b^
Quality of life					
FDD-QoL^1^	258	48.32 (15.27)	51.45 (15.20)	45.09 (14.72)	**0.001** ^a^
Activities^2^	270	53.34 [40.63]	59.38 [41.29]	45.31 [39.17]	**<0.001** ^c^
Anxiety^2^	266	55.00 [38.13]	55.00 [41.25]	50.00 [40.00]	0.403^c^
Diet^2^	265	37.50 [37.50]	41.67 [44.17]	33.33 [37.50]	**0.003** ^c^
Sleep^2^	265	75.00 [41.67]	75.00 [33.33]	66.67 [33.33]	**0.019** ^c^
Discomfort^2^	264	38.89 [25.00]	43.75 [27.78]	36.11 [23.61]	**0.002** ^c^
Health perception^2^	262	37.50 [29.17]	40.00 [33.33]	33.33 [22.92]	**0.021** ^c^
Disease coping^2^	261	41.67 [33.33]	41.67 [33.33]	33.33 [29.17]	0.173^c^
Stress^2^	262	41.67 [41.67]	50.00 [41.67]	41.67 [41.67]	0.553^c^
SF-36					
Physical QoL^1^	250	40.44 (9.83)	42.99 (9.77)	37.64 (9.15)	**<0.001** ^a^
Mental QoL^2^	250	39.29 [21.02]	39.89 [22.06]	38.54 [19.89]	0.781^c^

^1^Mean (standard deviation). ^2^Median [interquartile range]. ^a^*t*-test. ^b^*χ*^2^ test. ^c^Mann–Whitney *U* test. ^∗^Most of the data of the column with FGID patients in total were shown similarly in Berens et al. [20]. ^∗∗^All patients with >1 FGID diagnoses had two FGIDs.

**Table 3:** Health care utilization and work-related impairment.

	Effective *n*	FGID patients in total^∗^ (*n* = 271) [20]	Patients with 1 FGID diagnosis (*n* = 141)	Patients with >1 FGID diagnoses (*n* = 130)^∗∗^	*p* value
Appointments in the FGID clinic (1 year)^2^	268	2.00 [2.00]	2.00 [2.00]	2.00 [2.00]	0.958^c^
*n* = 1, *n* (%)	268	122 (45.52)	62 (44.93)	60 (46.15)	0.840^c^
*n* = 2, *n* (%)	268	76 (28.36)	40 (28.99)	36 (27.69)	0.814^c^
*n* = 3, *n* (%)	268	39 (14.55)	22 (15.94)	17 (13.07)	0.506^c^
*n* ≥ 4, *n* (%)	268	31 (11.57)	14 (10.14)	17 (13.07)	0.453^c^
Appointment in the psychosomatic outpatient clinic of Heidelberg University, *n* (%)	271	82 (30.26)	40 (28.37)	42 (32.31)	0.481^b^
Medical appointments in the last 4 weeks^2^	259	2.00 [1.00]	1.00 [1.00]	2.00 [1.80]	0.496^c^
*n* = 1, *n* (%)		71 (27.41)	38 (29.00)	33 (25.78)	0.561^b^
*n* = 2, *n* (%)		70 (27.03)	32 (24.43)	38 (29.69)	0.341^b^
*n* ≥ 3, *n* (%)		64 (24.71)	32 (24.43)	32 (25.00)	0.915^b^
Psychotherapeutic treatment, *n* (%)	262	139 (53.05)	76 (55.47)	63 (50.40)	0.411^b^
Currently, *n* (%)		70 (26.72)	33 (24.09)	37 (29.60)	0.314^b^
Previously, *n* (%)		69 (26.34)	43 (31.39)	26 (20.80)	0.052^b^
Current medication for anxiety, depression, or stress, *n* (%)	259	78 (30.12)	40 (29.85)	38 (30.40)	0.923^b^
Previous inpatient stay, *n* (%) (lifetime)	268	43 (16.04)	21 (15.22)	22 (16.92)	0.704^b^
Psych. acute care hospital, *n* (%)		27 (10.07)	13 (9.42)	14 (10.77)	0.714^b^
Psych. rehabilitation hospital, *n* (%)		9 (3.36)	5 (3.62)	4 (3.08)	1.000^b^
Work-related impairment due to GI symptoms in the last year (IBS-SSS)					
Weeks absent from work (absenteeism)^2^	155	2.00 [6.00]	1.00 [3.00]	3.00 [10.00]	**0.002** ^c^
Weeks at work suffering (presenteeism)^2^	83	25.00 [48.00]	12.00 [31.00]	38.00 [44.00]	**0.003** ^c^

^1^Mean (standard deviation). ^2^Median [interquartile range]. ^a^*t*-test. ^b^*χ*^2^ test. ^c^Mann–Whitney *U* test. ^∗^Most of the data of the column with FGID patients in total were shown similarly in Berens et al. [20]. ^∗∗^All patients with >1 FGID diagnoses had two FGIDs.

## Data Availability

Data is available on reasonable request.

## Abbreviations

FD: Functional dyspepsia
FGID: Functional gastrointestinal disorder
GI: Gastrointestinal
IBS: Irritable bowel syndrome.
